# Cholangioscopy-assisted endoscopic radial incision for benign biliary stricture

**DOI:** 10.1055/a-2652-3564

**Published:** 2025-09-26

**Authors:** Yawei Bi, Bo Zhang, Yi Yao, Fang Liu, Enqiang Linghu, Ningli Chai

**Affiliations:** 1 104607 Department of Gastroenterology, The First Medical Center of Chinese PLA General Hospital, Beijing, China

Endoscopic therapy has emerged as first-line management for most benign biliary stricture (BBS) patients 1 . However, endoscopic management of high-grade strictures remains challenging. This is primarily due to difficulty in cannulating the strictured segment with conventional guidewires and secondarily to technical difficulty in achieving precise dilation at the stenotic site. Herein, we performed endoscopic radial incision under direct choledochoscopic visualization for a stenotic bile duct, achieving precise therapeutic intervention for BBS.

A 69-year-old male with a history of cholecystectomy 7 years prior presented with recurrent jaundice and fever over the past six months. Magnetic resonance cholangiopancreatography demonstrated stenosis of the proximal common bile duct (CBD) ( Fig. 1 ). During endoscopic retrograde cholangiopancreatography, selective cannulation was achieved, but the guidewire could not be advanced beyond the proximal CBD. Subsequent cholangioscopy revealed a fibrotic stricture at the CBD, with direct visual biopsy confirming inflammatory ductal changes ( Fig. 2 ). Given the severe luminal narrowing, conventional fluoroscopy-guided wire-based balloon/bougie dilation was deemed technically unfeasible and high-risk. Our team developed a needle-type electrosurgical knife (outer diameter: 1.0 mm, working tip length: 1.5 mm) designed for the working channel of a peroral cholangioscope ( Fig. 3 ). We subsequently performed a cholangioscopy-assisted radial incision under direct vision to address this BBS.

**Fig. 1:**
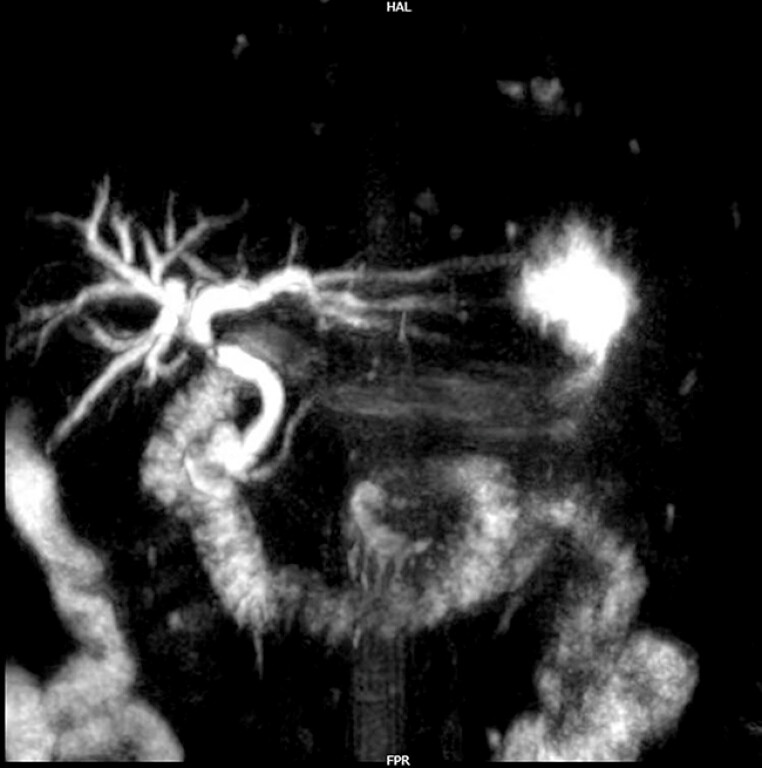
Preoperative MRCP image of the patient depicting biliary stricture.

**Fig. 2:**
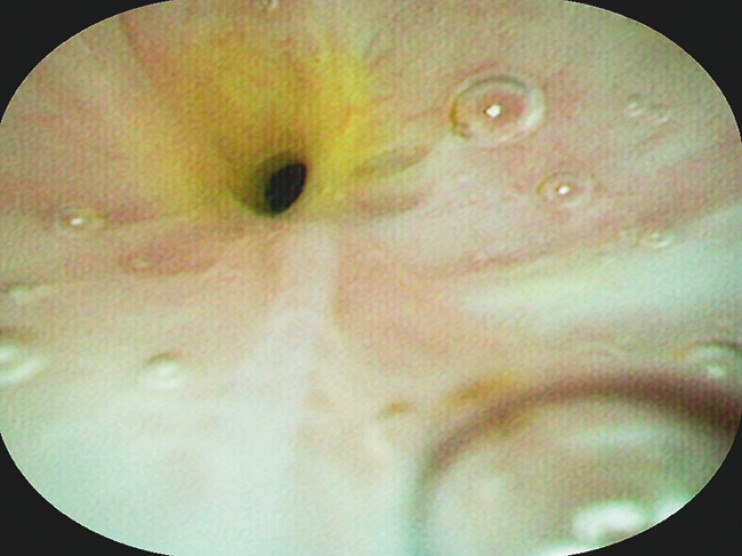
Choledochoscopic view demonstrating scar-like stricture in CBD.

**Fig. 3:**
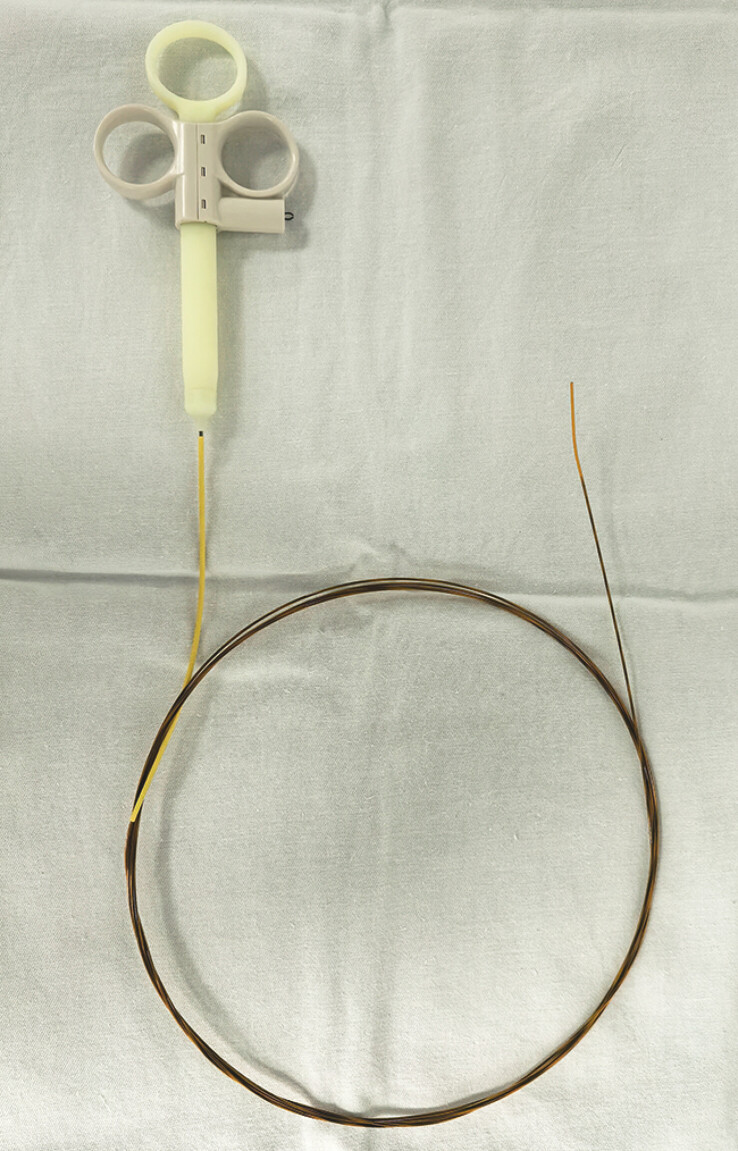
The needle-type electrosurgical knife designed for the working channel of a peroral cholangioscope.

A cholangioscope (EyeMax, 9 Fr; Micro-Tech) was advanced into the CBD, revealing a fibrotic stricture in the CBD under direct endoscopic visualization. The specifically designed needle-knife was then advanced through the working channel and used to incise the CBD stricture under direct vision without evidence of bleeding or perforation ( Fig. 4 ). Following incision, a rounded calculus dislodged from above the stricture, suggesting impaction as the cause of the patientʼs recent recurrent jaundice and fever ( Fig. 5 ). Post-incision, the cholangioscope traversed the stricture and visualized a suture line clearly observed proximal to the stricture in the CBD ( Video 1 ). Subsequently, two biliary stents (Cotton-Leung, 10 Fr, 8 cm, COOK) were placed to dilate the treated stricture. Postoperatively, serum amylase and lipase levels were mildly elevated at 6 hours but normalized by 24 hours, and the patient was discharged on day 3 without adverse events.

**Fig. 4:**
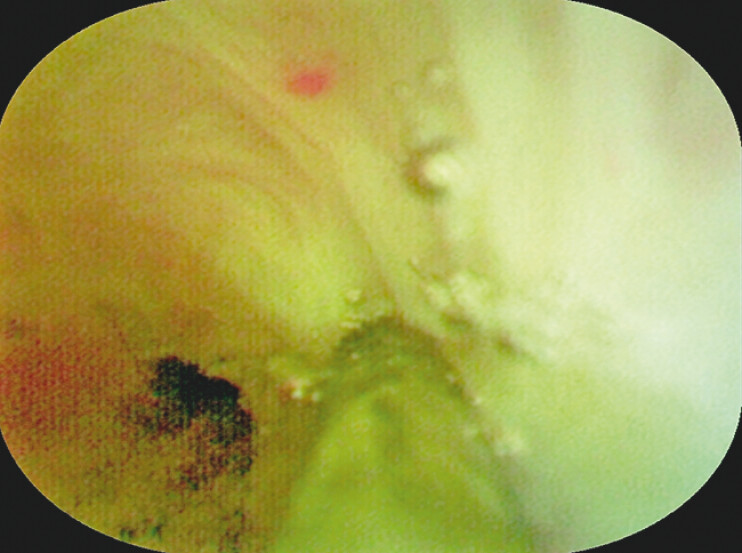
Electrosurgical incision under direct cholangioscopic vision guidance for biliary stricture.

**Fig. 5:**
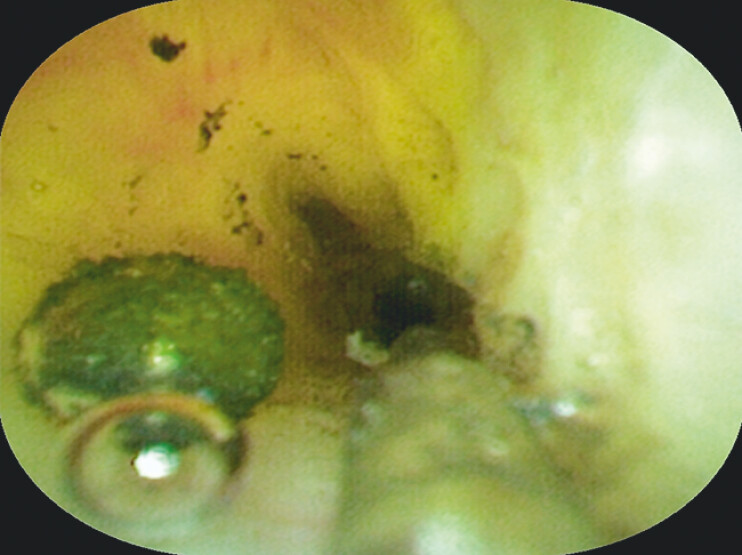
A rounded calculus dislodged from above the CBD stricture.

Cholangioscopy-assisted endoscopic radial incision for benign biliary stricture.Video 1

We present the case of electrosurgical incision under direct cholangioscopic vision for BBS, offering a novel minimally invasive endoscopic approach for such challenging cases.

Endoscopy_UCTN_Code_TTT_1AR_2AI

## Notes

Endoscopy E-Videos https://eref.thieme.de/e-videos
*E-Videos* is an open access online section of the journal *Endoscopy* , reporting on interesting cases and new techniques in gastroenterological endoscopy. All papers include a high-quality video and are published with a Creative Commons CC-BY license. Endoscopy E-Videos qualify for HINARI discounts and waivers and eligibility is automatically checked during the submission process. We grant 100% waivers to articles whose corresponding authors are based in Group A countries and 50% waivers to those who are based in Group B countries as classified by Research4Life (see: https://www.research4life.org/access/eligibility/ ). This section has its own submission website at https://mc.manuscriptcentral.com/e-videos .
